# Predictors of long-term outcomes with hip resurfacing

**DOI:** 10.4103/0019-5413.77128

**Published:** 2011

**Authors:** Mohit Bhandari, Sarah Culgin

**Affiliations:** *Department of Orthopaedics, McMaster University, Hamilton, Ontario, Canada*

## CITATION

Treacy RB, McBryde CW, Shears E, Pynsent PB. Birmingham Hip Resurfacing: A minimum follow-up of ten years. J Bone Joint Surg Br. 2011;93:27-33.

## RESEARCH QUESTION

To determine the survival and the radiological and functional outcome of a series of consecutive Birmingham hip resurfacing procedures after a minimum follow-up of 10 years.

## MATERIALS AND METHODS

Patients who had been enrolled in the 5-year follow-up of the first consecutive single-surgeon series of Birmingham hip resurfacing procedures done between August 1997 and May 1998 were followed up at a minimum of 10 years post hip resurfacing surgery. Standardized pre and postoperative regimes and a posterior approach were used in all 144 hips. At the 10-year follow-up patients completed an Oxford hip score (OHS; scored according to Pynsent, Adams, and Disney), a UCLA activity score, and a questionnaire regarding revision surgeries. A standardized anteroposterior radiograph of the pelvis was also taken. Patients not able to complete the visit in person were contacted by phone and had pelvic radiographs taken at their local hospital. No patients were lost to follow-up. Location of surgery and reason for revision were obtained from the attending surgeon or hospital for all reported revision surgeries. All deaths were analyzed for any relationship to the hip resurfacing procedure. Radiographs were analyzed by a blinded independent researcher using OsiriX open-source software (OsiriX foundation, Geneva, Switzerland). The inclination angle of the acetabular component and the femoral component to the femoral shaft were measured, i.e., the obtuse angle between the center of the proximal femoral canal and the center of the femoral component. The presence of a radiolucent line >2 mm width in any of the three zones described by Amstuts *et al*. was considered as loosening of the femoral component. A radiolucent line >2 mm in any of the two or more zones described by DeLee and Charnley was recorded as acetabular loosening. Any osteolysis around the femoral and/or acetabular component was recorded. A reduction in the minimum width of the femoral neck on the 10-year follow-up radiograph was considered as thinning of the femoral neck if the reduction was >10% in comparison to the initial postoperative radiograph.

## RESULTS

Of the 130 patients (144 hip resurfacing surgeries), 9 died during the study period, 10 hips were revised and leaving 111 patients (124 hips) available for review. None of the deaths were considered related to the hip resurfacing procedure. The survival with death as the endpoint was 94.9% (95% CI: 91.1 to 98.6) at 10 years, and 93.5% (95% CI: 89.2 to 97.6) and 95.5% (95% CI 91.8 to 99.0) with endpoints of revision for any reason and aseptic revision, respectively. With revision for any reason as the endpoint, Cox’s proportional hazard analysis for survival identified head size and gender to be significantly associated with revision (*P*=.0006 and *P*=.0002, respectively). Kaplan-Meier survival for male patients was 98.0% (95% CI: 95.2 to 100) and was 97.7% (95% CI: 94.6 to 100) for patients with femoral component >50 mm. Risk of revision was increased 1.14 times per year with every 4 mm decrease in femoral component size, and increased 5.78 times per year if the patient was female. For the 88.3% (98 patients) who completed the modified OHS, the median modified OHS was 4.2% (IQR: 0% to 19%). For the 81.1% (90 patients) who completed the UCLA activity score, the mean was 7.0% (IQR: 5.0% to 8.0%). Of the 77 radiographs available for review, none showed either femoral or acetabular osteolysis or loosening of the femoral or acetabluar components. The mean acetabluar inclination angle was 49° (95% CI: 49 to 50) and the mean component to shaft angle was 141° (95% CI: 140 to 142).

## RELEVANCE TO ORTHOPEDIC PRACTICE

The results of this 10-year follow-up indicate that the metal-on-metal Birmingham hip resurfacing procedure can be considered an acceptable alternative to conventional total hip replacement for male patients requiring a high level of functioning and low risk of revision for at least 10 years. Although the majority of failures in the reported series occurred in female patients, follow-up of a large cohort of females would be necessary before making any conclusions regarding the suitability of this hip resurfacing procedure in women.

## EXPERT OPINION

This well conducted study raises important questions about failure rates associated with head size and gender. As the authors suggest, a sample size of 130 patients is inadequate to make any definite conclusions about true risk for females and patients with smaller femoral head components.

